# The Impact of Urate‐Lowering Therapy in Post‐Myocardial Infarction Patients: Insights From a Population‐Based, Propensity Score‐Matched Analysis

**DOI:** 10.1002/cpt.2473

**Published:** 2021-11-17

**Authors:** Chi‐Jung Tai, Chin‐Chung Wu, Kun‐Tai Lee, Tzyy‐Guey Tseng, Hui‐Chun Wang, Fang‐Rong Chang, Yi‐Hsin Yang

**Affiliations:** ^1^ Department of Family Medicine Kaohsiung Medical University Hospital Kaohsiung Medical University Kaohsiung Taiwan; ^2^ Graduate Institute of Natural Products College of Pharmacy Kaohsiung Medical University Kaohsiung Taiwan; ^3^ Department of Family Medicine Pingtung Hospital Ministry of Health and Welfare Pingtung Taiwan; ^4^ Cardiovascular Center Kaohsiung Medical University Hospital Kaohsiung Medical University Kaohsiung Taiwan; ^5^ Department of Marine Biotechnology and Resources National Sun Yat‐Sen University Kaohsiung Taiwan; ^6^ Drug Development and Value Creation Research Center Kaohsiung Medical University Kaohsiung Taiwan; ^7^ Department of Medical Research Kaohsiung Medical University Hospital Kaohsiung Medical University Kaohsiung Taiwan; ^8^ School of Pharmacy College of Pharmacy Kaohsiung Medical University Kaohsiung Taiwan; ^9^ National Institute of Cancer Research National Health Research Institutes Tainan Taiwan

## Abstract

The role of urate‐lowering therapy (ULT) for the primary prevention of cardiovascular (CV) events has been widely discussed, but its evidence for the secondary prevention of myocardial infarction (MI) is limited. Therefore, we conduct a population‐based, propensity score‐matched cohort study to investigate the CV outcomes among patients with post‐MI with and without ULT. A total of 19,042 newly diagnosed in‐hospital patients with MI were selected using the Taiwan National Health Insurance Database between January 1, 2005, and December 31, 2016. After 1:1 propensity score matching with covariates, patients with MI with (*n* = 963) and without (*n* = 963) ULT were selected for further analysis. The primary outcome was the all‐cause mortality and the secondary outcomes were composite CV outcomes, including hospitalization for recurrent MI, stroke, heart failure, and cardiac arrhythmias. ULT users were associated with lower all‐cause mortality (adjusted hazard ratio (adjHR), 0.67; 95% confidence interval (CI), 0.51–0.87) compared to the ULT nonusers. In addition, ULT users had a significantly lower risk of recurrent MI, which needed revascularization by percutaneous coronary intervention or coronary artery bypass grafting (adjHR, 0.67; 95% CI, 0.53–0.86) than the ULT nonusers. The primary and secondary outcomes were not different between patients with post‐MI who received uricosuric agents and xanthine oxidase inhibitors. The anti‐inflammatory effect of ULT plays an essential role in MI management. From a real‐world setting, this study shows that ULT is associated with the lower risk of all‐cause mortality in patients with post‐MI. In addition, the result shows the possible lower incidence of repeat revascularization procedures in the ULT users.

**Study Highlights**

**WHAT IS THE CURRENT KNOWLEDGE ON THE TOPIC?**

☑ Urate‐lowering therapy (ULT) has shown its benefit on the primary prevention of cardiovascular (CV) events.

**WHAT QUESTION DID THIS STUDY ADDRESS?**

☑ Can ULT decrease all‐cause mortality and CV events in patients post myocardial infarction (MI)?

**WHAT DOES THIS STUDY ADD TO OUR KNOWLEDGE?**

☑ ULT is associated with the lower risk of all‐cause mortality, lower incidence of repeat revascularization procedures, and lower risk of stroke hospitalization.

**HOW MIGHT THIS CHANGE CLINICAL PHARMACOLOGY OR TRANSLATIONAL SCIENCE?**

☑ We suppose that the result might encourage the use of ULT in patients with post‐MI.

Inflammation is widely believed as a treatment target in atherosclerosis and coronary artery diseases (CADs).^1^, ^2^ Serum uric acid (UA), a metabolite of purines, is considered a significant inflammatory biomarker for cardiovascular disease (CVD).^3^ Therefore, urate‐lowering therapy (ULT) is generally used to decrease cardiovascular (CV) risks and mortality effectively.^4^

Exploring the mechanisms, excessive intake of purine diet, overproduction of urate, and underexcretion of urate through the kidneys may lead to hyperuricemia.^5^ Currently, there are two primary types of ULT for hyperuricemia. One is the xanthine oxidase inhibitors (XOIs), such as allopurinol and febuxostat, which reduce the endogenous production of UA.^6^ The other consists of uricosuric agents, including benzbromarone, probenecid, or sulfinpyrazone, which enhance the renal clearance of UA.^7^ In addition to mitigating CV risks, ULT has been used for managing gout,^8^ reducing stone events in patients with hyper‐uricosuric urolithiasis,^9^ reducing CV events in patients with heart failure (HF),^10^ and preventing chronic kidney disease progression.^11^

To the best of our knowledge, most clinical studies have focused on the role of ULT in the primary prevention of CV risks and mortality. For the secondary prevention of CAD, the effect of ULT in patients with myocardial infarction (MI) and chronic coronary syndromes is unclear. In the 2019 European Society of Cardiology (ESC) guideline for the diagnosis and management of chronic coronary syndromes, the evidence regarding the effect of allopurinol in reducing clinical events in CVD was limited.^12^ Rajendra *et al*. reported that high‐dose allopurinol (600 mg/day) significantly improved endothelium‐dependent vasodilation in patients with stable CAD.^13^ In addition, a randomized, placebo‐controlled crossover trial of 65 patients with CAD or stable chronic angina pectoris showed that high‐dose allopurinol (600 mg/day) increased the time to ST depression during exercise, total exercise time, and time to chest pain.^14^ However, the effect of uricosuric agents or febuxostat on chronic coronary syndrome was not discussed in the 2019 ESC guideline. Therefore, the purpose of this study was to assess the effect of ULT on mortality and CV outcomes in patients with post‐MI using the Taiwan population‐based database.

## METHODS

### Data source

This retrospective cohort study was conducted using the Taiwan National Health Insurance (NHI) Database and Taiwan Death Registry (TDR). The single‐payer NHI program was launched in Taiwan in 1995 and has enrolled more than 99% of the 23 million people in Taiwan. The Taiwan NHI database contains claim records of beneficiaries, including demographic data, inpatient records, outpatient prescriptions, and expenditure for healthcare services. Data management and statistical analyses were performed at the Health and Welfare Data Science Center (HWDC), which is managed by the Department of Statistics, Ministry of Health and Welfare, Taiwan. The HWDC provides government databases to conduct the research. For the sake of privacy protection, all personal identifications are encrypted, and only authorized researchers are permitted to process databases in a designated area. Moreover, only statistical results can be used for publications. This study was approved by the Institutional Review Board of Antai Medical Care Cooperation Antai‐Tian‐Sheng Memorial Hospital (protocol number, TSMHIRB‐17‐028‐C0; approval date, March 6, 2017).

### Enrollment and exposures

**Figure 1:**
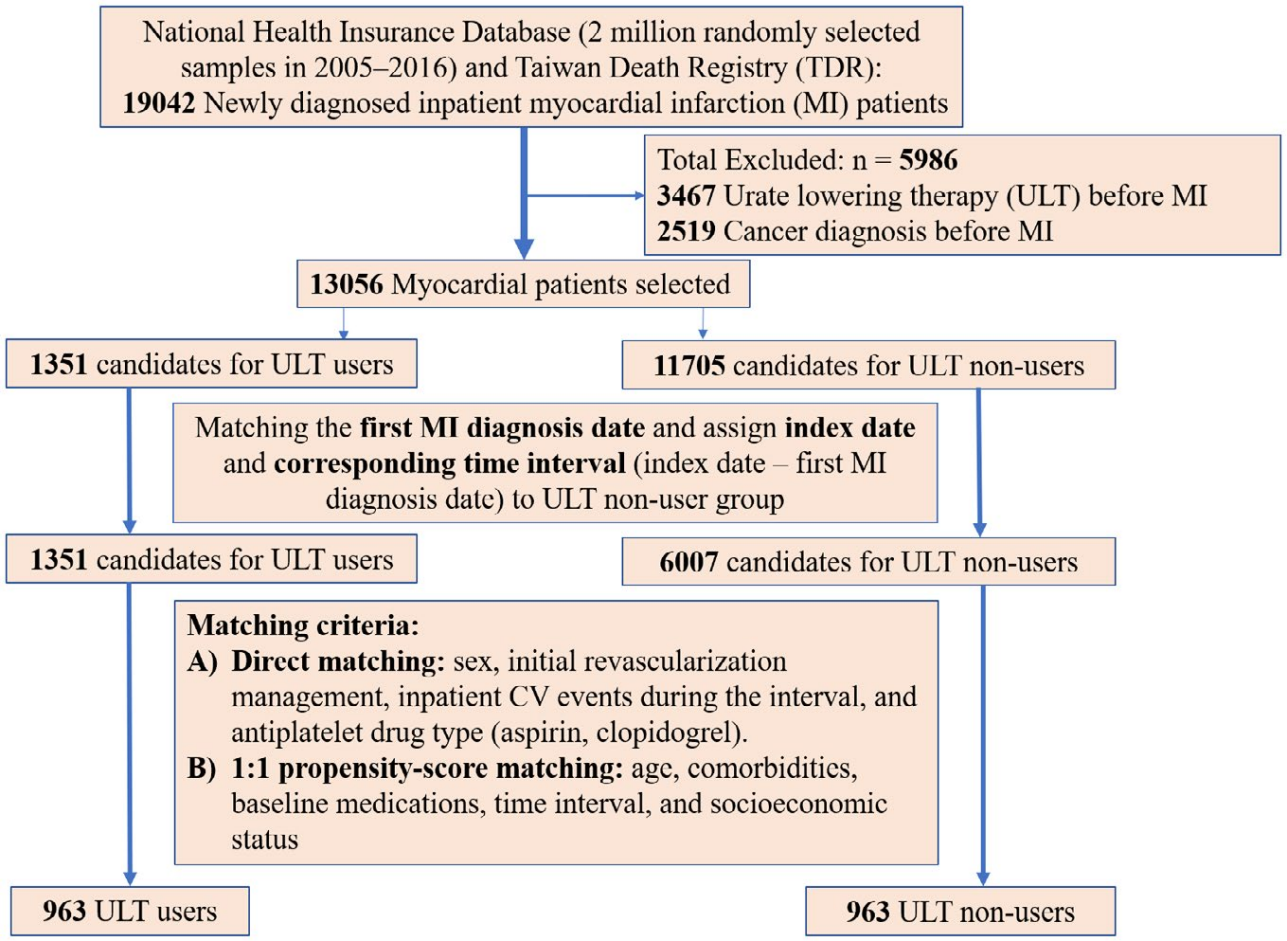
Flowchart of the study design, matching criteria, and allocation of the study subjects. CV, cardiovascular. [Colour figure can be viewed at wileyonlinelibrary.com]

**Figure 2:**
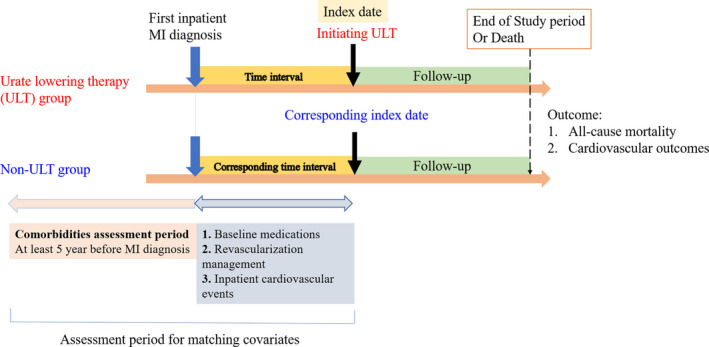
Study design, time sequences, assessment periods, and the follow‐up period of the study. MI, myocardial infarction. [Colour figure can be viewed at wileyonlinelibrary.com]

We used a longitudinal cohort of the Taiwan NHI Database from 2000 to 2016, which comprised a randomly sampled representative database of 2 million people from all living NHI enrollees in 2005. The sampling indicated that patients with newly diagnosed MI before 2005 might have a survival bias. Therefore, we included patients aged 20 years or older with a new diagnosis of in‐hospital MI (International Classification of Diseases Revision, Ninth Revision, Clinical Modification (ICD‐9‐CM) code: 410; International Classification of Diseases Revision, Tenth Revision, Clinical Modification (ICD‐10‐CM): I21 and I22) between January 1, 2005, and December 31, 2016 (**Figure** **1**).^15^ The ICD‐9‐CM codes were used between 2005 and 2015, and the ICD‐10‐CM codes were used in 2016. The clinical data of the enrolled participants between 2000 and 2004 was used to assess comorbidities over a 5‐year baseline period (**Figure** **2**).

The first hospitalization for MI was considered the first inpatient MI diagnosis date (**Figure** **2**). The ULT group included patients who had ever used XOIs (allopurinol or febuxostat) or uricosuric agents (benzbromarone, probenecid, or sulfinpyrazone). Target medications were identified according to the Anatomical Therapeutic Chemical classification system and the corresponding drug codes in the NHI Database. For the ULT group, the date of ULT initiation after MI was the index date, and the end of follow‐up date was defined as the earliest date of the following: 60 days after the last prescription of the ULT,^16^, ^17^ date of death, and the last day of the cohort. This ensured that the outcomes in the ULT group were related to ULT use.

Out of 19,042 patients with newly diagnosed MI, 3,467 patients who received ULT before MI were excluded because the variation in the duration of ULT uses before the index MI increased the selection bias. In addition, we excluded 2,519 patients who were diagnosed with cancer before MI (**Figure** **1**). A total of 1,351 and 11,705 patients with MI were treated with ULT and without ULT, respectively.

### Assigning the index date to the non‐ULT group

Immortal time refers to a span of time in the observation or follow‐up period of a cohort during which the outcome under study could not have occurred.^18^ Immortal time bias usually occurs in the user and nonuser study design, wherein the participants, especially in the nonuser group, have to remain event‐free until the start of exposure to be classified as exposed. Studies with immortal time bias have overestimated treatment effects.

In our study, the immortal time was defined as the interval from the first MI diagnosis date to the index date. Because there was no initiating date for ULT use in the non‐ULT group, assigning an appropriate index date to the non‐ULT group was essential to account for possible immortal time bias. First, we performed a 1:6 matching of the first inpatient MI diagnosis date between the two groups and assigned the index date and the corresponding time interval (from the first MI diagnosis date to the index date) to the non‐ULT group. Second, ULT nonusers were excluded if their follow‐up time was less than the assigned time interval. After this step, we included 1,351 patients with MI treated with ULT and 6,007 patients with MI without ULT (**Figure** **1**). This process minimized the possibility of immortal time bias in our study.

### Matching strategy

To minimize the effect of bias and confounders between the ULT and non‐ULT groups, we performed the following matching strategy to balance the covariates between the ULT and non‐ULT groups. Because we only have patients’ with MI ICD‐9‐CM and ICD‐10‐CM codes, it is not feasible to evaluate the MI type and severity. Instead, we directly matched the revascularization management, including heparinization, percutaneous coronary intervention (PCI), and coronary artery bypass graft (CABG) surgery, which were identified by specific treatment codes (PCI: 33076B, 3077B, and 33078B; and CABG: 68023B, 68024B, and 68025B) in inpatient medical claim orders during the time interval between the first inpatient MI diagnosis and index date (**Figure** **2**).^19^ In addition, we directly matched the sex and antiplatelet drug type (aspirin, clopidogrel, or dual‐antiplatelet) between the two groups. Because it was possible to have inpatient CV events, including recurrent MI, HF, CVD, and cardiac arrhythmias (supraventricular tachycardia, ventricular tachycardia, atrial fibrillation, and atrial flutter), during the time interval between the first MI diagnosis date and index date, we also directly matched these events between the two groups. Direct matching of these covariates could make the type and severity of MI in the two groups similar.

Furthermore, the propensity score (PS) was calculated with covariates, including age, concomitant comorbidities, baseline medications, time interval, and socioeconomic status (**Figure** **2** **)**. Concomitant comorbidities included hypertension, HF, diabetes mellitus with or without complications, chronic obstructive pulmonary disease, chronic kidney, liver and CVDs, and gout. Defining the comorbidities required at least 3 times diagnoses of the specific ICD‐9‐CM or ICD‐10‐CM codes from outpatient data in the NHI database before the first MI diagnosis date (**Figure** **2**).

Apart from antiplatelet drugs, we also considered the following medications: angiotensin converting enzyme inhibitors (ACEIs), angiotensin receptor blockers (ARBs), calcium channel blockers, ß‐blockers, statin, and other lipid‐lowering agents (fibrates, bile acid sequestrant resins, and nicotinic acids), oral anti‐diabetic drugs, insulin, diuretics, and colchicine. The baseline medications were defined as medications used between the time interval of the first MI diagnosis date and the index date (**Figure** **2**).

Finally, we generated a 1:1 PS‐matched cohort for our outcome analysis.^20^ We used Greedy nearest neighbor matching without replacement. We specified the caliper width of 0.25, which indicated that the difference in PS between the treated unit and its matching control unit must be less than or equal to 0.25. The direct and PS matching were conducted using the PSMATCH procedure provided by SAS (SAS Institute Inc., Cary, NC, USA). Standardized mean differences were calculated to compare the distribution of baseline covariates between the ULT and the non‐ULT groups after PS matching.^21^ A previous study suggested that a standardized mean difference above 0.1 denoted meaningful imbalance in the baseline covariates.

### Clinical outcomes

The primary outcome was all‐cause mortality. All‐cause mortality was identified in the TDR database, which included death date and cause of death. The secondary outcomes were composite CV outcomes, including CV death, hospitalization for recurrent MI, HF, stroke, and cardiac arrhythmias (supraventricular tachycardia, ventricular tachycardia, atrial fibrillation, and atrial flutter). Because the different revascularization methods implied the severity and mechanism of recurrent MI, we further evaluated the recurrent MI with revascularization by PCI or CABG, and by heparinization.^19^ The occurrence of recurrent MI with revascularization was defined as an admission to a hospital for MI and the reception of heparinization, PCI, or CABG surgery during hospitalization.

We further evaluated the effects of XOIs and uricosuric agents in the ULT group. Apart from the previous definition of the end of follow‐up, patients were also censored when they began combinations or switched between both ULT types. The matching strategy and primary and secondary outcomes of drug comparison were the same as those in the previous setting.

### Statistical analyses

Baseline characteristics were described as the mean ± SD for continuous variables and the number and percentage for categorical variables. The Kaplan–Meier method was used to compare the two groups for time‐to‐event analysis. Furthermore, we performed the log‐rank test and Wilcoxon test to detect the difference in cumulative mortality curve between the ULT and non‐ULT groups.^22^ The Wilcoxon test weighed the group differences by sample size at each time period, whereas the log rank test used the same weighting for the group differences at each time period. Both tests evaluated the robustness of the treatment’s effect on overall survival.

The Cox proportional hazards models were constructed to estimate adjusted hazard ratios (adjHRs) and 95% confidence intervals (CIs) of all‐cause mortality. The adjHRs and 95% CIs of secondary outcomes were calculated using the subdistribution proportional hazard regression, which considered death as a competing event.^23^ We adjusted the models for all possible confounders and the use of major CV medications after the index date as covariates.^24^ All of the analyses were conducted using SAS version 9.4. Statistical significance was set at *P* < 0.05.

### Sensitivity analyses

Because the sample size of this study was small after the enrollment process and matching, the robustness of the results might be questioned.^25^ Therefore, we performed a bootstrapping approach as a sensitivity analysis. Initially, using the SURVEYSELECT procedure, with an unrestricted random sampling method in SAS, we generated 1,000 bootstrap samples.^26^ Then, the bootstrap adjHRs and 95% CIs were estimated from bootstrap samples using Cox regression and subdistribution proportional hazard regression.^27^ The bootstrapping approach demonstrated a more robust nonparametric estimate of the adjHRs and 95% CIs, which avoided parametric assumptions.

## RESULTS

### Baseline characteristics

After matching through our protocol, the ULT (*n* = 963) and non‐ULT (*n* = 963) groups were well balanced at the baseline (**Table** **1**, **Figure** **S1**). The mean follow‐up was 2.9 ± 2.7 (median, 2.1) years. The average time of patients using ULT was 2.5 ± 2.7 (median, 1.3) years. The mean age was 65.6 ± 13.3 (median, 65.9) and 65.5 ± 13.3 (median, 65.4) years in the ULT and non‐ULT groups, respectively. The time interval between the first MI diagnosis date and index date was 2.2 ± 2.3 (median, 1.4) years in the ULT group and 2.1 ± 2.1 (median, 1.4) years in the non‐ULT group.

**Table 1:** Demographic characteristics of patients post‐MI with or without ULT after 1:1 PS matching

	ULT (+) *n* = 963	ULT (−) *n* = 963	Standardized difference^a^
Age mean ± SD, years	65.6 ± 13.3	65.5 ± 13.8	0.009
Male sex	742 (77.1%)	742 (77.1%)	< 0.001
Interval,^b^ mean ± SD, years	2.2 ± 2.3	2.1 ± 2.1	0.036
Revascularization management for initial MI			
PCI	700 (72.7%)	700 (72.7%)	< 0.001
CABG	95 (9.9%)	95 (9.9%)	< 0.001
Heparinization	132 (13.7%)	132 (13.7%)	< 0.001
Inpatient cardiovascular events during first MI date and index date			
Recurrent MI	117 (12.1%)	117 (12.1%)	< 0.001
Heart failure	148 (15.4%)	148 (15.4%)	< 0.001
Cerebrovascular disease	23 (2.4%)	23 (2.4%)	< 0.001
Cardiac arrhythmias	25 (2.6%)	25 (2.6%)	< 0.001
Antiplatelet drugs			
Dual antiplatelet	772 (80.1%)	772 (80.1%)	< 0.001
Aspirin	891 (92.5%)	891 (92.5%)	< 0.001
Clopidogrel	812 (84.3%)	812 (84.3%)	< 0.001
No antiplatelet drugs	32 (3.3%)	32 (3.3%)	< 0.001
Medications			
ACEI/ARBs	686 (71.2%)	707 (73.4%)	0.047
Calcium channel blockers	433 (45.0%)	441 (45.8%)	0.017
ß‐blockers	740 (76.8%)	759 (78.8%)	0.044
Statins	685 (71.1%)	681 (70.7%)	0.009
Other lipid lowering agents	168 (17.4%)	162 (16.8%)	0.018
OADs	368 (38.2%)	407 (42.3%)	0.085
Insulin	162 (16.8%)	159 (16.5%)	0.009
Diuretics	568 (59.0%)	582 (60.4%)	0.031
Colchicine	147 (15.3%)	137 (14.2%)	0.031
Comorbidities			
Hypertension	691 (71.8%)	705 (73.2%)	0.031
Heart failure	141 (14.6%)	147 (15.3%)	0.017
Cerebrovascular disease	218 (22.6%)	221 (22.9%)	0.007
DM without complications	370 (38.4%)	398 (41.3%)	0.060
DM with complications	167 (17.3%)	161 (16.7%)	0.017
COPD	370 (38.4%)	356 (37.0%)	0.030
Cardiac arrhythmias	150 (15.6%)	170 (17.7%)	0.055
Gastric or peptic ulcers	355 (36.9%)	371 (38.5%)	0.034
Chronic kidney disease	134 (13.9%)	139 (14.4%)	0.015
Chronic liver disease	190 (19.7%)	212 (22.0%)	0.057
Gout	230 (23.9%)	236 (24.5%)	0.015
Educational level			
Elementary school and below	473 (49.1%)	471 (48.9%)	0.037
Junior high	179 (18.6%)	188 (19.5%)	
Senior high	193 (20.0%)	181 (18.8%)	
College and above	118 (12.3%)	123 (12.8%)	
Marital status			
Unmarried	101 (10.5%)	103 (10.7%)	0.029
Married	715 (74.2%)	703 (73.0%)	
Divorce/death of spouse	147 (15.3%)	157 (16.3%)	
Living area			
Northern part	453 (47.0%)	443 (46.0%)	0.028
Middle part	133 (13.8%)	141 (14.6%)	
Southern part	320 (33.2%)	324 (33.6%)	
Others	57 (5.9%)	55 (5.7%)	
Enrollee category			
< 28,000 NTD	695 (72.2%)	695 (72.2%)	< 0.001
≥ 28,000 NTD	268 (27.8%)	268 (27.8%)	

ACEI, angiotensin converting enzyme inhibitor; ARB, angiotensin receptor blocker; CABG, coronary artery bypass grafting; COPD, chronic obstructive pulmonary disease; DM, diabetes mellitus; MI, myocardial infarction; NTD, New Taiwan Dollar; OADs, oral anti‐diabetic agents; PCI, percutaneous coronary intervention; PS, propensity score; ULT, urate‐lowering therapy.

^a^ The Standardized mean difference above 0.1 might denote meaningful imbalance in the baseline covariates.

^b^ Interval is the time interval between first MI diagnosis date and index date.

Out of all subjects, 72.7% received PCI, 9.9% received CABG, and 13.7% received heparinization as the initial revascularization management for index MI. About 92.5% of the patients with MI took aspirin and 84.3% took clopidogrel. Moreover, ~ 80.1% of patients with MI took dual antiplatelets. Only 3.3% of the patients with MI did not take any antiplatelet drugs (**Table** **1**). The results showed that most of the patients enrolled in this study received basic antiplatelet treatment.

The top three other baseline medications were ß‐blockers (76.8%–78.8%), ACEI/ARBs (71.2%–73.4%), and statins (70.7%–71.1%). Before matching, the prevalence of colchicine use was significantly different between the ULT (32.7%) and non‐ULT (3.0%) groups (**Table** **S1**). This result was consistent with that of the ULT group, which had a higher prevalence of gout (28.8%) than the non‐ULT group (15.4%). The difference in colchicine use between the two groups indicated a heavier disease burden in the ULT group before matching. After matching, patients in the ULT (15.3%) and non‐ULT (14.2%) groups had similar colchicine administration rates.

The top three most frequent comorbidities among the patients with MI were hypertension (71.8%–73.2%), diabetes mellitus without complications (38.4%–41.3%), and chronic obstructive pulmonary disease (37.0%–38.4%; **Table** **1**). The detailed demographics of the ULT and non‐ULT groups before and after matching were shown in **Table** **S1** and **Table** **1**, respectively.

### ULT vs. non‐ULT

**Figure 3:**
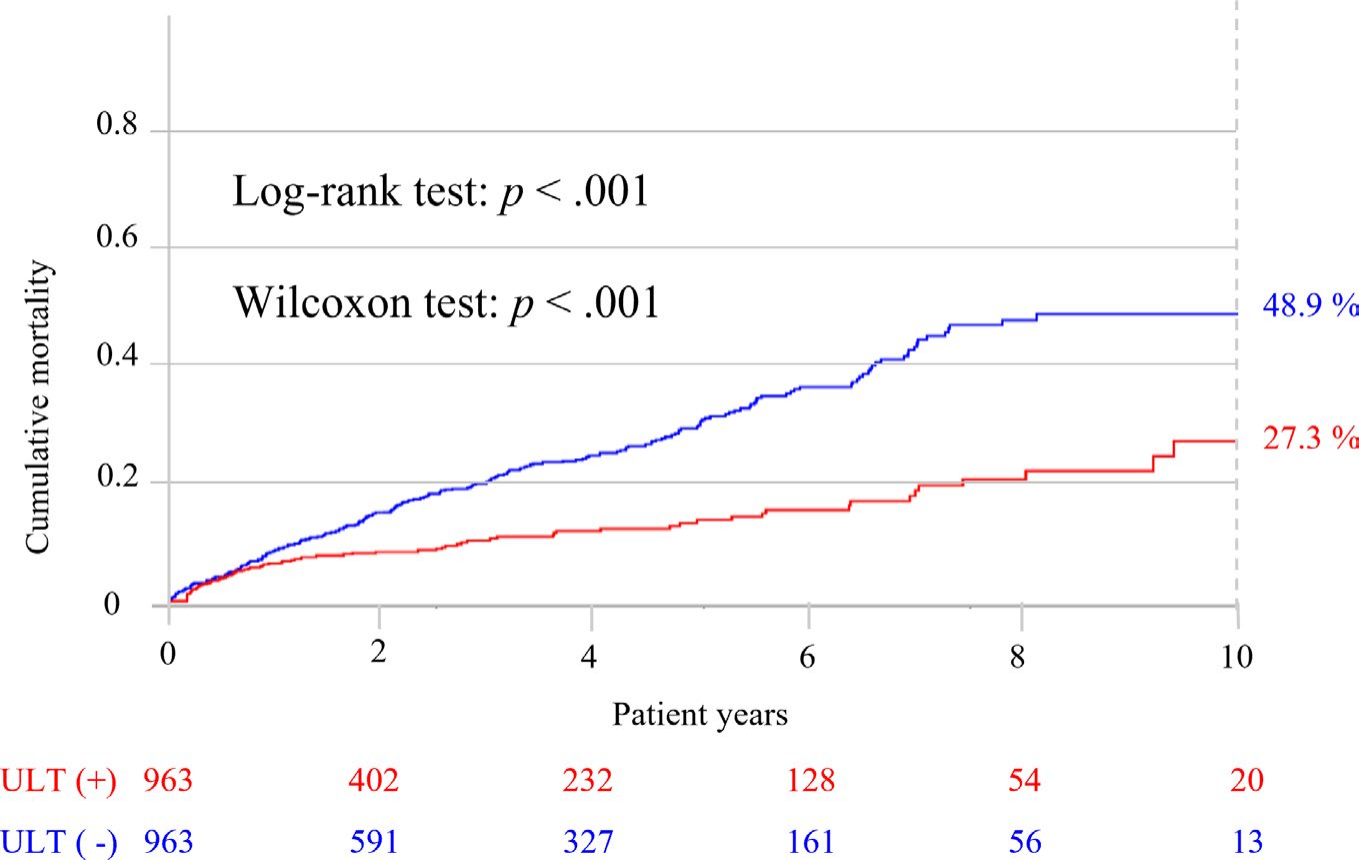
Kaplan–Meier analysis of all‐cause mortality between the ULT and Non‐ULT groups. ULT, urate lowering therapy. [Colour figure can be viewed at wileyonlinelibrary.com]

The ULT group had a significantly lower risk of all‐cause mortality than the non‐ULT group, with an adjHR of 0.67 (95% CI, 0.51–0.87; bootstrap adjHR, 0.66; bootstrap 95% CI, 0.48–0.90; **Table** **2**). The cumulative all‐cause mortality was 27.3% in the ULT group and 48.9% in the non‐ULT group, and the Kaplan–Meier survival curves began to separate after the first year of follow‐up (**Figure** **3**). Both the log rank test (*P *< 0.001) and Wilcoxon test (*P* < 0.001) showed the treatment effect of ULT on overall survival.

**Table 2:** Comparison of all‐cause mortality and cardiovascular outcomes between patients with and without ULT

	ULT (+)	ULT (−)	Adjusted HR^a^ (95% CI)	*P* value^a^	Bootstrap adjusted HR (bootstrap 95% CI)^b^
	(*n* = 963)	(*n* = 963)			
Primary end points					
All‐cause mortality	86 (8.9%)	245 (25.4%)	0.67 (0.51–0.87)	0.003^*^	0.66 (0.48–0.90)
Secondary end points					
Composite CV outcomes^c^	315 (32.7%)	376 (39.0%)	0.90 (0.76–1.08)	0.26	0.90 (0.75–1.10)
Recurrent MI with revascularization by PCI or CABG	138 (14.3%)	188 (19.5%)	0.67 (0.53–0.86)	0.001^*^	0.69 (0.53–0.90)
Recurrent MI with revascularization by heparinization	190 (19.7%)	245 (25.4%)	0.79 (0.64–0.97)	0.02^*^	0.81 (0.64–1.01)
Heart failure hospitalization	160 (16.6%)	157 (16.3%)	1.25 (0.97–1.60)	0.09	1.40 (1.08–1.83)
Stroke hospitalization	35 (3.6%)	69 (7.1%)	0.60 (0.38–0.96)	0.03^*^	0.58 (0.35–0.95)
Cardiac arrhythmias hospitalization^d^	47 (4.9%)	68 (7.1%)	0.86 (0.55–1.34)	0.51	0.91 (0.57–1.51)

CABG, coronary artery bypass grafting; CI, confidence interval; CV, cardiovascular; HR, hazard ratio; MI, myocardial infarction; PCI, percutaneous coronary intervention; ULT, urate‐lowering therapy.

^a^ Hazard ratio (HR) and *P* value of all‐cause mortality was estimated by Cox proportional hazard regression. HRs and *P* values of composite CV outcomes, recurrent MI, heart failure, stroke, and cardiac arrhythmias were calculated by competing risk analysis. All HRs and *P* values were adjusted for all covariates listed in Table 1. ^*^ *P* value < 0.05.

^b^ Bootstrap adjusted HRs and 95% CIs were estimated using 1000 bootstrap samples. Details were described in the Method section.

^c^ Composite CV outcomes included cardiovascular death, admission due to recurrent myocardial infarction, stroke, heart failure, and cardiac arrhythmias.

^d^ Cardiac arrhythmias comprises supraventricular tachycardia, ventricular tachycardia, atrial fibrillation, and atrial flutter.

ULT use was associated with a lower risk of repeat MI revascularization procedures by PCI or CABG (adjHR, 0.67; 95% CI, 0.53–0.86; bootstrap adjHR, 0.69; bootstrap 95% CI, 0.53–0.90). However, ULT use showed equivocal results in heparinization (adjHR, 0.79; 95% CI, 0.53–0.86; bootstrap adjHR, 0.81; bootstrap 95% CI, 0.64–1.01). In addition, ULT users also had a lower risk of stroke hospitalization with an adjHR of 0.60 (95% CI, 0.38–0.96; bootstrap adjHR, 0.58; bootstrap 95% CI, 0.35–0.95). However, ULT use did not show a protective effect in HF and cardiac arrhythmia hospitalizations (**Table** **2**).

### XOIs vs. uricosuric agents

From 1,351 ULT user candidates (**Figure** **1**), we excluded 321 ULT users, who had an initial combination of uricosuric agent and XOI at the index date and divided them into the uricosuric agent group and the XOI group. After matching, 364 patients with MI were treated with uricosuric agents and XOIs for further analysis (**Table** **S2**). The results showed that the primary and secondary outcomes were not different between the two groups (**Table** **3**), indicating that there was no priority for uricosuric agents or XOIs in patients with post‐MI.

**Table 3:** Comparison of all‐cause mortality and cardiovascular outcomes between XOI and uricosuric agent users

	Uricosuric agent users	XOI users	Adjusted HR^a^ (95% CI)	*P* value^a^
	(*n* = 364)	(*n* = 364)		
Primary end points				
All‐cause mortality	64 (17.6%)	71 (19.5%)	1.10 (0.77–1.57)	0.62
Secondary end points				
Composite CV outcomes^b^	157 (43.1%)	149 (40.9%)	1.15 (0.91–1.45)	0.24
Recurrent MI with revascularization by PCI or CABG	73 (20.1%)	71 (19.5%)	1.14 (0.83–1.56)	0.42
Recurrent MI with revascularization by heparinization	89 (24.5%)	86 (23.6%)	1.14 (0.85–1.55)	0.38
Heart failure hospitalization	64 (17.6%)	57 (15.7%)	1.23 (0.85–1.78)	0.27
Stroke hospitalization	22 (6.0%)	30 (8.2%)	0.70 (0.41–1.22)	0.21
Cardiac arrhythmias hospitalization^c^	25 (6.9%)	25 (6.9%)	1.27 (0.70–2.31)	0.42

CABG, coronary artery bypass grafting; CI, confidence interval; CV, cardiovascular; HR, hazard ratio; MI, myocardial infarction; PCI, percutaneous coronary intervention; XOI, xanthine oxidase inhibitor.

^a^ Hazard ratio (HR) and *P* value of all‐cause mortality was estimated by Cox proportional hazard regression. HRs and *P* values of composite CV outcomes, recurrent MI, heart failure, stroke, and cardiac arrhythmias were calculated by competing risk analysis. All HRs and *P* values were adjusted for all covariates listed in **Table** **S3**. *P value < 0.05.

^b^ Composite CV outcomes included CV death, admission due to recurrent nonfatal MI, stroke, heart failure, and cardiac arrhythmias.

^c^ Cardiac arrhythmias comprises supraventricular tachycardia, ventricular tachycardia, atrial fibrillation, and atrial flutter.

## DISCUSSION

Although ULT is often used to prevent CV outcomes in patients with gout or hyperuricemia in clinical settings,^28^ there have been several debates about the role of serum UA levels in patients with MI. Previous studies showed that an elevated UA level was associated with higher sudden death and in‐hospital mortality in patients with MI undergoing PCI.^29^ In addition, Lazaros *et al*. showed that the peak UA level was an independent predictor of both 30‐day and 1‐year mortalities of patients with MI.^30^ Lazzeri *et al*. also showed that UA was an independent predictor of 1‐year mortality in patients with MI with an HR of 1.26.^31^ This study provided real‐world evidence that suggest ULT was associated with a lower risk of all‐cause mortality in patients with post‐MI during long‐term management. Interestingly, this study also showed that ULT was associated with a lower incidence of recurrent MI that required revascularization procedures for PCI, CABG, and heparinization. It is worth exploring whether ULT has different effects on ST‐elevation MI (STEMI) and non‐STEMI, and its underlying mechanism.

A meta‐analysis showed that hyperuricemia was associated with a significantly higher risk of stroke incidence (relative risk, 1.41; 95% CI, 1.05–1.76).^32^ Yen *et al*. showed that ULT users had a lower risk of hospitalized stroke (adjHR, 0.52; 95% CI, 0.39–0.70) in patients with gout.^33^ Our study further demonstrated that ULT use was associated with hospitalized stroke in patients with post‐MI. In short‐term follow‐ups, Kojima *et al*. reported that the UA level was associated with Killip’s classification suggestive of left ventricular (LV) HF in patients with MI.^34^ Our study showed an equivocal result in the impact of ULT on HF hospitalization; where the adjHR in the parametric estimate was 1.25 (95% CI, 0.97–1.60), but the bootstrap adjHR in the nonparametric estimate was 1.40 (1.08–1.83; **Table** **2**). Currently, some clinical trials are aimed to evaluate the effect of ULT on HF prevention.^35^, ^36^

### Possible mechanisms

Apart from the benefit directly from urate lowering, other beneficial effects of ULT in patients with post‐MI may originate from additional mechanisms. For example, allopurinol improved the endothelial function, which resulted in the improvement of the peripheral vasodilator capacity and blood flow.^37^ In particular, high‐dose allopurinol (600 mg/day) regressed LV mass, reduced the LV end‐systolic volume, and improved the endothelial function in patients with ischemic heart disease (IHD) and LV hypertrophy.^38^ In addition, allopurinol decreased arterial stiffness, and the progression of carotid intima‐media thickening.^39^ The ongoing ALL‐HEART study is aimed to evaluate the effect of high‐dose allopurinol (600 mg/day) in patients with IHD.^40^ Benzbromarone possessed the ability as a scavenger of free radicals and reduced oxidative stress in the endothelial cells induced by UA and angiotensin II.^41^ Moreover, ULT was found to lower the blood pressure.^42^

### Strength and limitations

The strength of this study is that it used a large population‐based cohort and considered the potential of confounding factors in the Taiwan NHI database, such as sex, revascularization management type, antiplatelet drug, baseline medications, concomitant comorbidities, and socioeconomic status. The results of this study contribute to understanding the impact of ULT in patients with MI from a real‐world setting.

Although our study generated essential and exciting findings, the results should be interpreted with caution. First, although we used multiple strategies to minimize confounding, the current observational study may have residual confounding factors and cannot prove the causality. For example, some clinical or laboratory data were not available in the Taiwan NHI databases, including healthy behaviors, UA levels, cardiac echo findings, electrocardiography, and body mass indexes. In addition, we only evaluated the common baseline medications because it was impossible to evaluate all concurrent medications. Furthermore, the possible confounding factors may have a greater effect on comparisons between uricosuric agents and XOIs because of the small number of cases. Second, the population included in the present analysis was primarily Asian and, therefore, may not represent the results for White patients or other populations.

Third, it is difficult to consider all issues of time‐varying exposure‐related problems, including treatment episodes construction, time‐varying confounders, cumulative exposure and latency, and treatment switching, not only in pharmacoepidemiology but also clinical trial study.^43^ In our study, the definition of end of follow‐up date and the adjustment for the post‐index date of primary CV minimizes the bias. Although some may suggest to evaluate the dose‐response relationship between ULT and patients with post‐MI, it has another issue because the authors had to look forward in time to determine the cumulative dose.

Fourth, the median time interval between the first MI diagnosis date and index date was ~ 2.2 years. Therefore, the results of this study could only support that ULT use was beneficial to patients with long‐term MI. The effect of ULT on acute coronary syndrome was not clear. Fifth, we did not further evaluate the effect of each XOI or uricosuric agent. In the CARES study, febuxostat was noninferior to allopurinol with respect to the rates of adverse CV events in patients with gout and major CV co‐existing conditions.^44^ The ongoing Febuxostat vs. Allopurinol Streamlined Trial (FAST) trial may provide evidence for Febuxostat and allopurinol in preventing non‐fatal MI, non‐fatal stroke, or CV death in the near future.^45^ Further studies comparing the effects of the different types of XOIs and uricosuric agents on patients are required to improve clinical applications. Finally, this study did not provide evidence for specific stratifications among age, sex, and comorbidities due to the limited number of cases in each stratum.

In conclusion, the anti‐inflammatory effect of ULT plays an essential role in MI management. This study shows that ULT is associated with a lower risk of all‐cause mortality in patients with post‐MI based on a real‐world database. In addition, the results show a possibly lower incidence of repeat revascularization procedures in ULT users. Furthermore, ULT users are associated with a lower risk of stroke hospitalization. However, there is no prior choice between the XOIs and uricosuric agents. We suppose that the real‐world evidence from this study will be valuable for considering the use of ULT in patients with post‐MI. However, further clinical trials are required to evaluate these assumptions.

## FUNDING

This work was supported by the grants from Ministry of Science and Technology (MOST), Taiwan (108‐2320‐B‐037‐022‐MY3, 108‐2811‐B‐037‐511, and 109‐2927‐I‐037‐502) to Fang‐Rong Chang, by MOST (103‐2320‐B‐037‐007‐MY3) to Hui‐Chun Wang, and by National Health Research Institutes (CA‐110‐PP‐09) to Yi‐Hsin Yang. The sponsor played no role in conduct of the study, analysis and interpretation of data, preparation, review, or approval of the manuscript and decision to submit the manuscript for publication.

## CONFLICT OF INTEREST

The authors declared no competing interests for this work.

## AUTHOR CONTRIBUTIONS

C.‐J.T., C.‐C.W., T.‐G.T., and Y.‐H.Y. wrote the manuscript. C.‐J.T., K.‐T.L., F.‐R.C., and Y.‐H.Y. designed the research. C.‐J.T., H.‐C.W., and Y.‐H.Y. performed the research. C.‐J.T. and Y.‐H.Y. analyzed the data.

## Supporting information

Supplementary Material
