# Supplementary material for: The Impact of Urate‐Lowering Therapy in Post‐Myocardial Infarction Patients: Insights From a Population‐Based, Propensity Score‐Matched Analysis
Source: Clin Pharmacol Ther. 2021 Nov 17;111(3):655–63. doi: 10.1002/cpt.2473 (PMC9298734; doi:10.1002/cpt.2473)
Supplement: Supplementary file 1 — Supplementary Material [file CPT-111-655-s001.docx]

| **Table S1.** Demographic characteristics of patients post myocardial infarction with or without urate-lowering therapy (ULT) before 1:1 propensity-score (PS) matching | | |
| --- | --- | --- |
|  | **ULT (+)**  ***n* = 1351** | **ULT (-)**  ***n* = 6007** |
| Age, (Mean±SD), years | 66.1±13.5 | 64.9±14.1 |
| Male sex | 1046 (77.4%) | 4293 (71.5%) |
| Interval ^a^, (Mean±SD), years | 2.3±2.4 | 1.8±2.0 |
| Revascularization management for initial MI | | |
| PCI | 941 (69.7%) | 4001 (66.6%) |
| CABG | 148 (11.0%) | 393 (6.5%) |
| Heparinization | 205 (15.2%) | 1050 (17.5%) |
| Inpatient cardiovascular events during first MI date and index date | | |
| Recurrent MI | 210 (15.5%) | 593 (9.9%) |
| Heart failure | 272 (20.1%) | 494 (8.2%) |
| Cerebrovascular disease | 59 (4.4%) | 282 (4.7%) |
| Cardiac arrhythmias ^b^ | 78 (5.8%) | 228 (3.8%) |
| Antiplatelet drugs | | |
| Dual antiplatelet | 1043 (77.2%) | 4022 (67.0%) |
| Aspirin | 1223 (90.5%) | 4975 (82.8%) |
| Clopidogrel | 1116 (82.6%) | 4316 (71.8%) |
| No antiplatelet drugs | 55 (4.1%) | 738 (12.3%) |
| Baseline medications | | |
| ACEI/ARBs | 1000 (74.0%) | 3657 (60.9%) |
| Calcium channel blockers | 636 (47.1%) | 2031 (33.8%) |
| ß-blockers | 1043 (77.2%) | 3860 (64.2%) |
| Statins | 956 (70.8%) | 3585 (59.7%) |
| Other lipid lowering agents | 227 (16.8%) | 724 (12.1%) |
| OADs | 521 (38.6%) | 1849 (30.8%) |
| Insulin | 247 (18.3%) | 607 (10.1%) |
| Diuretics | 852 (63.1%) | 1982 (33.0%) |
| Colchicine | 442 (32.7%) | 179 (3.0%) |
| Comorbidities | | |
| Hypertension | 991 (73.4%) | 3761 (62.6%) |
| Heart failure | 221 (16.4%) | 813 (13.5%) |
| Cerebrovascular disease | 325 (24.1%) | 1528 (25.4%) |
| DM without complications | 544 (40.3%) | 2235 (37.2%) |
| DM with complications | 241 (17.8%) | 908 (15.1%) |
| COPD | 515 (38.1%) | 2205 (36.7%) |
| Cardiac arrhythmias | 238 (17.6%) | 1020 (17.0%) |
| Gastric or peptic ulcers | 515 (38.1%) | 2331 (38.8%) |
| Chronic kidney disease | 193 (14.3%) | 771 (12.8%) |
| Chronic liver disease | 267 (19.8%) | 1223 (20.4%) |
| Gout | 389 (28.8%) | 924 (15.4%) |
| Educational level | | |
| Elementary school and below | 677 (50.1%) | 2888 (48.1%) |
| Junior High | 255 (18.9%) | 1043 (17.4%) |
| Senior High | 271 (20.1%) | 1283 (21.4%) |
| College and above | 148 (11.0%) | 793 (13.2%) |
| Marital status | | |
| Unmarried | 136 (10.1%) | 643 (10.7%) |
| Married | 1006 (74.5%) | 4352 (72.4%) |
| Divorce/Death of spouse | 209 (15.5%) | 1012 (16.8%) |
| Living area | | |
| Northern part | 646 (47.8%) | 2908 (48.4%) |
| Middle part | 189 (14.0%) | 932 (15.5%) |
| Southern part | 434 (32.1%) | 1821 (30.3%) |
| Others | 82 (6.1%) | 346 (5.8%) |
| Enrollee category | | |
| < 28,000 NTD | 979 (72.5%) | 4307 (71.7%) |
| ≥ 28,000 NTD | 372 (27.5%) | 1700 (28.3%) |
| ^a^ Interval is the time interval between first MI diagnosis date and index date.  ^b^ Cardiac arrhythmias comprises supraventricular tachycardia, ventricular tachycardia, atrial fibrillation, and atrial flutter.  ACEI, angiotensin converting enzyme inhibitor; ARB, angiotensin receptor blocker; CABG, coronary artery bypass grafting; COPD, chronic obstructive pulmonary disease; DM, diabetes mellitus; MI, myocardial infarction; NTD, New Taiwan Dollar; OADs, oral anti-diabetic agents; PCI, percutaneous coronary intervention; SD, standard deviation; ULT, urate-lowering therapy. | | |

| **Table S2.** Demographic Characteristics of Myocardial Infarction Patients with the Use of Uricosuric Agents or Xanthine Oxidase Inhibitor after 1:1 propensity-score (PS) matching | | | |
| --- | --- | --- | --- |
|  | **Uricosuric agent users**  ***n* = 364** | **XOI users**  ***n* = 364** | **Standardized difference** ^a^ |
| Age, (Mean±SD), years | 65.0±12.9 | 66.5±13.1 | 0.115 |
| Male sex | 302 (83.0%) | 302 (83.0%) | < 0.001 |
| Interval ^b^ | 2.4±2.5 | 2.3±2.3 | 0.037 |
| Revascularization management for initial MI | | | |
| PCI | 285 (78.3%) | 285 (78.3%) | < 0.001 |
| CABG | 20 (5.5%) | 20 (5.5%) | < 0.001 |
| Heparinization | 44 (12.1%) | 44 (12.1%) | < 0.001 |
| Inpatient cardiovascular events during first MI date and index date | | | |
| Recurrent MI | 45 (12.4%) | 45 (12.4%) | < 0.001 |
| Heart failure | 52 (14.3%) | 52 (14.3%) | < 0.001 |
| Cerebrovascular disease | 6 (1.7%) | 6 (1.7%) | < 0.001 |
| Cardiac arrhythmias | 7 (1.9%) | 7 (1.9%) | < 0.001 |
| Antiplatelet drugs | | | |
| Dual antiplatelet | 314 (86.3%) | 314 (86.3%) | < 0.001 |
| Aspirin | 343 (94.2%) | 343 (94.2%) | < 0.001 |
| Clopidogrel | 326 (89.6%) | 326 (89.6%) | < 0.001 |
| No antiplatelet drugs | 9 (2.4%) | 9 (2.4%) | < 0.001 |
| Baseline medications | | | |
| ACEI/ARBs | 271 (74.5%) | 276 (75.8%) | 0.031 |
| Calcium channel blockers | 159 (43.7%) | 171 (47.0%) | 0.066 |
| ß-blockers | 285 (78.3%) | 284 (78.0%) | 0.007 |
| Statins | 263 (72.3%) | 256 (70.3%) | 0.042 |
| Other lipid lowering agents | 71 (19.5%) | 55 (15.1%) | 0.118 |
| OADs | 142 (39.0%) | 132 (36.3%) | 0.057 |
| Insulin | 61 (16.8%) | 47 (12.9%) | 0.099 |
| Diuretics | 213 (58.5%) | 212 (58.2%) | 0.006 |
| Colchicine | 126 (34.6%) | 129 (35.4%) | 0.017 |
| Comorbidities | | | |
| Hypertension | 254 (69.8%) | 258 (70.9%) | 0.025 |
| Heart failure | 55 (15.1%) | 50 (13.7%) | 0.037 |
| Cerebrovascular disease | 73 (20.1%) | 74 (20.3%) | 0.006 |
| DM without complications | 144 (39.6%) | 137 (37.6%) | 0.039 |
| DM with complications | 64 (17.6%) | 64 (17.6%) | < 0.001 |
| COPD | 129 (35.4%) | 146 (40.1%) | 0.096 |
| Cardiac arrhythmias | 53 (14.6%) | 56 (15.4%) | 0.022 |
| Gastric or peptic ulcers | 127 (34.9%) | 131 (36.0%) | 0.023 |
| Chronic kidney disease | 49 (13.5%) | 47 (12.9%) | 0.016 |
| Chronic liver disease | 65 (17.9%) | 50 (13.7%) | 0.104 |
| Gout | 100 (27.5%) | 100 (27.5%) | < 0.001 |
| Educational level | | | |
| Elementary school | 165 (45.3%) | 179 (49.2%) | 0.126 |
| Junior High | 69 (18.9%) | 75 (20.6%) |  |
| Senior High | 85 (23.4%) | 68 (18.7%) |  |
| College | 45 (12.4%) | 42 (11.5%) |  |
| Marital status | | | |
| Unmarried | 36 (9.9%) | 27 (7.4%) | 0.092 |
| Married | 281 (77.2%) | 286 (78.6%) |  |
| Divorce/Death of spouse | 47 (12.9%) | 51 (14.0%) |  |
| Living area | | | |
| Northern | 160 (44.0%) | 168 (46.2%) | 0.060 |
| Middle | 61 (16.8%) | 54 (14.8%) |  |
| Southern | 117 (32.1%) | 116 (31.9%) |  |
| Others | 26 (7.1%) | 26 (7.1%) |  |
| Enrollee category | | | |
| < 28,000 NTD | 267 (73.4%) | 274 (75.3%) | 0.044 |
| ≥ 28,000 NTD | 97 (26.7%) | 90 (24.7%) |  |
| ^a^ The Standardized mean difference above 0.1 might denote meaningful imbalance in the baseline covariates.  ^b^ Interval is the time interval between first MI diagnosis date and index date.  ACEI, angiotensin converting enzyme inhibitor; ARB, angiotensin receptor blocker; CABG, coronary artery bypass grafting; COPD, chronic obstructive pulmonary disease; DM, diabetes mellitus; MI, myocardial infarction; NTD, New Taiwan Dollar; OADs, oral anti-diabetic agents; PCI, percutaneous coronary intervention; SD, standard deviation; ULT, urate-lowering therapy. | | | |


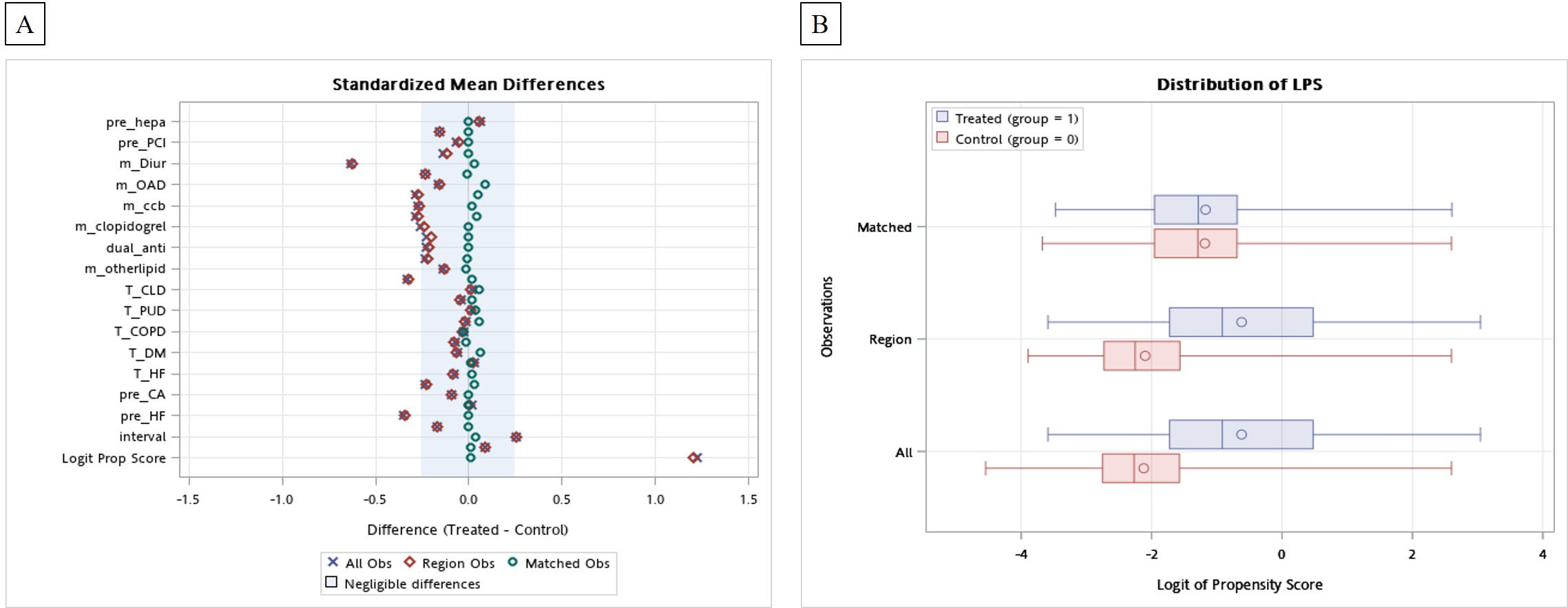


**Figure S1.** (A) Love Plot (B) Distribution of Logit of Propensity Score between ULT (treated) and non-ULT (control) group. ULT, urate lowering therapy.
